# Identification of novel Ack1-interacting proteins and Ack1 phosphorylated sites in mouse brain by mass spectrometry

**DOI:** 10.18632/oncotarget.20929

**Published:** 2017-09-15

**Authors:** Maria del Mar Masdeu, Beatriz G. Armendáriz, Anna La Torre, Eduardo Soriano, Ferran Burgaya, Jesús Mariano Ureña

**Affiliations:** ^1^ Department of Cell Biology, Faculty of Biology, University of Barcelona, Barcelona 08028, Spain; ^2^ Centro de Investigación Biomédica en Red sobre Enfermedades Neurodegenerativas (CIBERNED), ISCIII, 28031 Madrid, Spain; ^3^ Present address: Francis Crick Institute, Mill Hill Laboratory, Mill Hill, London NW7 1AA, United Kingdom; ^4^ Present address: Department of Cell Biology and Human Anatomy, University of California Davis, 95616 Davis, California, USA; ^5^ Vall d´Hebron Institute of Research, Barcelona 08035, Spain; ^6^ Institució Catalana de Recerca i Estudis Avançats (ICREA), Barcelona 08010, Spain

**Keywords:** tyrosine kinase, Ack1, central nervous system, development

## Abstract

Ack1 (activated Cdc42-associated tyrosine kinase) is a non-receptor tyrosine kinase that is highly expressed in brain. This kinase contains several protein-protein interaction domains and its action is partially regulated by phosphorylation. As a first step to address the neuronal functions of Ack1, here we screened mouse brain samples to identify proteins that interact with this kinase. Using mass spectrometry analysis, we identified new putative partners for Ack1 including cytoskeletal proteins such as Drebrin or MAP4; adhesion regulators such as NCAM1 and neurabin-2; and synapse mediators such as SynGAP, GRIN1 and GRIN3. In addition, we confirmed that Ack1 and CAMKII both co-immunoprecipitate and co-localize in neurons. We also identified that adult and P5 samples contained the phosphorylated residues Thr 104 and Ser 825, and only P5 samples contained phosphorylated Ser 722, a site linked to cancer and interleukin signaling when phosphorylated. All these findings support the notion that Ack1 could be involved in neuronal plasticity.

## INTRODUCTION

Protein phosphorylation is a key event of signaling cascades involved in cell proliferation, differentiation, adhesion or migration [1, 2]. It has been estimated that 30% of all proteins in the cell are phosphorylated [3, 4]. These phosphorylation events occur mostly on serine (Ser), threonine (Thr) and tyrosine (Tyr) residues in a ratio of 1800:200:1 [5, 6].

Ack1 is a cytoplasmic tyrosine kinase that contains several protein-protein interaction domains. From the N- to the C-terminal ends, the peptide sequence comprises the following: a sterile-alpha-motif (SAM), a catalytic tyrosine kinase domain that includes the major autophosphorylation residue of this protein (Tyr 284), a SH3 domain, a CRIB domain, and a long proline (Pro)-rich region that extends roughly along the C-terminal half of the polypeptide. Inside this long Pro-rich region lie additional protein-protein interaction domains, namely a Clathrin-binding domain (CBD), a sorting nexin 9-binding domain (SNX9), a WW-binding domain (WWBD), a Mig6 homology region (MHR) that includes an epidermal growth factor receptor (EGFR)-binding domain (EBD), and an ubiquitin-binding domain (Uba domain) [7, 8]. The presence of such a high number and variety of binding sites in Ack1 suggests that this molecule is involved in dynamic interactions with multiple partners. Phosphorylation events are crucial for the regulation of most kinases, and several phosphorylation sites of Ack1 have been described up to present. Specifically, the residues Thr 33 [9], Thr 133 and Thr 618 [10], Tyr 284 [11], Tyr 533 [12], Ser 724 [13], Tyr 518, Tyr 827, Tyr 859 and Tyr 872 [14], have been reported.

To date, Ack1 has been associated with actin cytoskeleton dynamics through binding to Cdc42 [15] and WASP [16]. Ack1 participation in EGF [14], 2007), PDGF [17], and neurotrophin signaling pathways [18] has also been described. Ack1 has been linked to endocytic processes through its binding to clathrin [19], and also to intracellular cascades related to cellular adhesion [20]. Furthermore, this tyrosine kinase has been related to the extension of lamellipodia and migration in particular cell types, and it may interact with various kinases such as Fyn [21]. In addition, Ack1 has been shown to be required for apoptosis induction by tumor necrosis factor [22], as well as in hepatocellular carcinoma progression [23], gastric tumorigenesis [24], glioma tumorigenesis [25], or prostate cancer [26]. At present, pharmacological inhibition of Ack1 is under development to be used as a treatment against cancer [27, 28].

Ack1 function depends partially on its state of phosphorylation [17], and the catalytic activity of the protein is enhanced by dimerization in a similar way to that of EGFR [8].

Ack1 expression is enriched 100 times in the Central Nervous System [29, 30] over other tissues. In adult animals, Ack1 mRNA is found mainly in the hippocampus, the cerebral cortex, the olfactory bulb, and the cerebellum [29]. Its expression during mouse brain development is also widespread, starting at embryonic day 10 (E10), with an enrichment in migrating and maturing neurons. Its expression is also high in the subventricular zone and in the rostral migratory stream, which both contain neurons destined to the olfactory bulb [30]. In a previous study, we showed that Ack1 positively regulates both neurotrophin-dependent and -independent neurite extension and branching [18] and that these functions are mediated by direct interaction of this kinase with the Trk family of neurotrophin receptors. In the mature brain, Ack1 is enriched in dendrites, dendritic spines, and presynaptic boutons, thereby suggesting that this protein contributes to neurotransmission [29].

At present, little is known about the molecular mechanisms involving Ack1 in neural function. Using Mass Spectrometry (MS) techniques, here we performed a proteomic screening to identify neuron-specific Ack1 partners. In addition, we applied MS to identify phosphorylation residues according to previous work [31–34]. This technique allowed us to identify phosphorylated peptides in a protein digest, along with the assignation of the identified peptides to specific proteins. Our data provide a collection of brain-specific Ack1 putative partners, as well as new Ack1 phosphorylation sites that may provide insights into the role of this kinase in neural function.

## RESULTS

### Protein extraction and LC-MS/MS and MALDI-TOF assays

**Figure 1:**
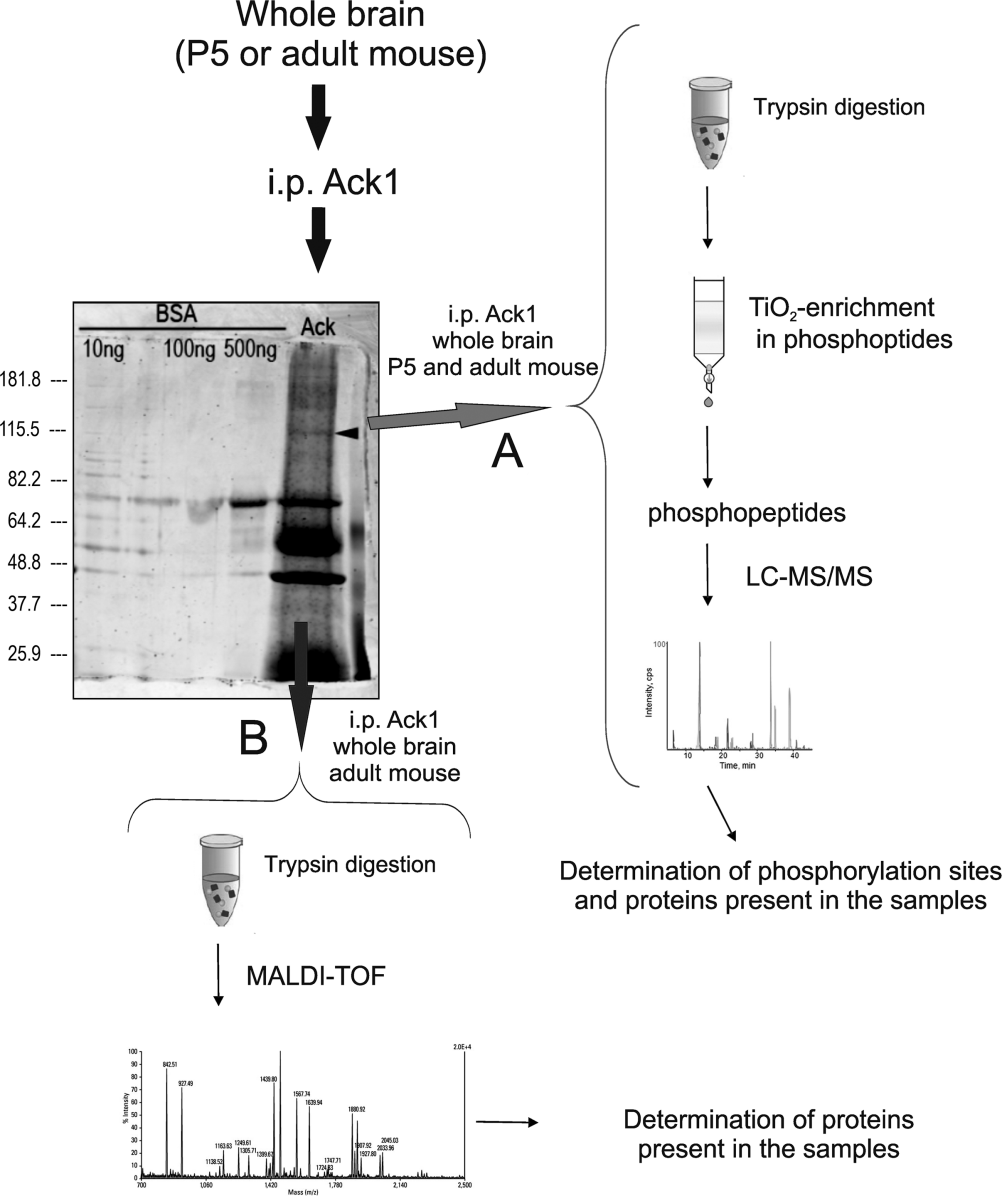
Protein extraction and LC-MS/MS analysis: Brains from mouse in each experimental condition were dissected, homogenized, and immunoprecipitated with antibodies against Ack1 The immunoprecipitation products were then loaded in a polyacrylamide gel and subjected to SDS-PAGE. The arrowhead on the gel points to Ack1. Afterwards, the gel was labeled with the fluorescent stain SyproRuby in approach (**A**) (red arrow) and with silver in (**B**) (blue arrow). The gel shown in the Figure corresponds to an adult sample immunoprecipitated and silver-stained, and the immunoprecipitation runs beside three lanes containing distinct amounts of bovine serum albumin-directed immunoprecipitates used as negative control. Adult and P5 samples showed the same pattern of staining at visual resolution. The experimental procedures were performed six times for adult samples and two times for P5 samples. Two different procedures were performed: (A) The first approach consisted of cutting the bands around the molecular weight of Ack1 (100 to 130 KDa approximately), avoiding the immunoglobulin bands. The former were then trypsinized and the tryptic digest was enriched in phosphopeptides with a column comprising titanium dioxide (TiO_2_) magnetic beads. Finally, the phosphopeptides obtained were analyzed by LC-MS/MS (right side of the figure). (B) The second approach consisted of silver staining plus band cutting and trypsinization of the eluted proteins, analogous to the previous procedure. The peptides obtained from the digestion were immediately analyzed by MALDI-TOF (lower part of the figure).

Here we identified a series of Ack1 phosphorylation sites and new putative binding partners by applying Ack1 immunoprecipitation in tandem with LC-MS/MS (Figure 1). The brain homogenates were treated and subjected to either LC-MS/MS procedures, as described in the Materials and Methods section (Figure 1). MALDI-TOF analysis was performed in our first assays and was considered for protein identification, although we decided not to consider this method for phosphopeptide evaluation. Thus, the most reliable MALDI-TOF-obtained partners are included in Supplementary Table 1 although phosphopeptide contents was only studied by LC-MS/MS.

### Proteins identified in the immunoprecipitated samples of Ack1

Ack1 immunoprecipitates were separated by SDS-PAGE and analyzed by LC-MS/MS to identify putative Ack1-interacting partners. A band covering the proteins weighing approximately 100 to 130 KDa was selected for these studies. On the basis of score values, we selected the most prevalent and reliable candidates (see the corresponding section in Materials and Methods). The number of putative partners was surprisingly high, and included more than 100 potential Ack1-interacting proteins (Supplementary Tables 2–3). Some of the proteins showing the highest fitting (Supplementary Table 1) are linked to cytoskeletal organization and dynamics, thereby suggesting that Ack1 modulates cell shape and cytoskeletal arrangement. Drebrin, an actin-interacting protein regulated in brain during development [35] and involved in plasticity and the regulation of spine structure [36] can be ascribed to this category, as can microtubule-associated proteins MAP 4 [37] and MAP 6 [38]. In addition, proteins associated with cell adhesion, such as NCAM 1 and the linker between actin cytoskeleton and cadherin receptors called Neurabin-2 [39] are listed. We also found proteins related to synaptic transmission, such as the NMDA receptor subunits GRIN1 and GRIN3 [40], and the neuron-specific GTPase-activating protein SynGAP [41]. Some proteins involved in cell signaling were also detected, such as CAMKII-α [42]. Of note, while some of these putative partners, such as drebrin, MAP4, MAP 6, and GRIN 1, were observed in adult as well as in P5 samples, others were detected exclusively in the former (SynGAP, GRIN 3 and CAMKII) or the latter (NCAM 1 among others) (Supplementary Table 1).

### Phosphorylated residues found in the Ack1 protein

**Figure 2:**
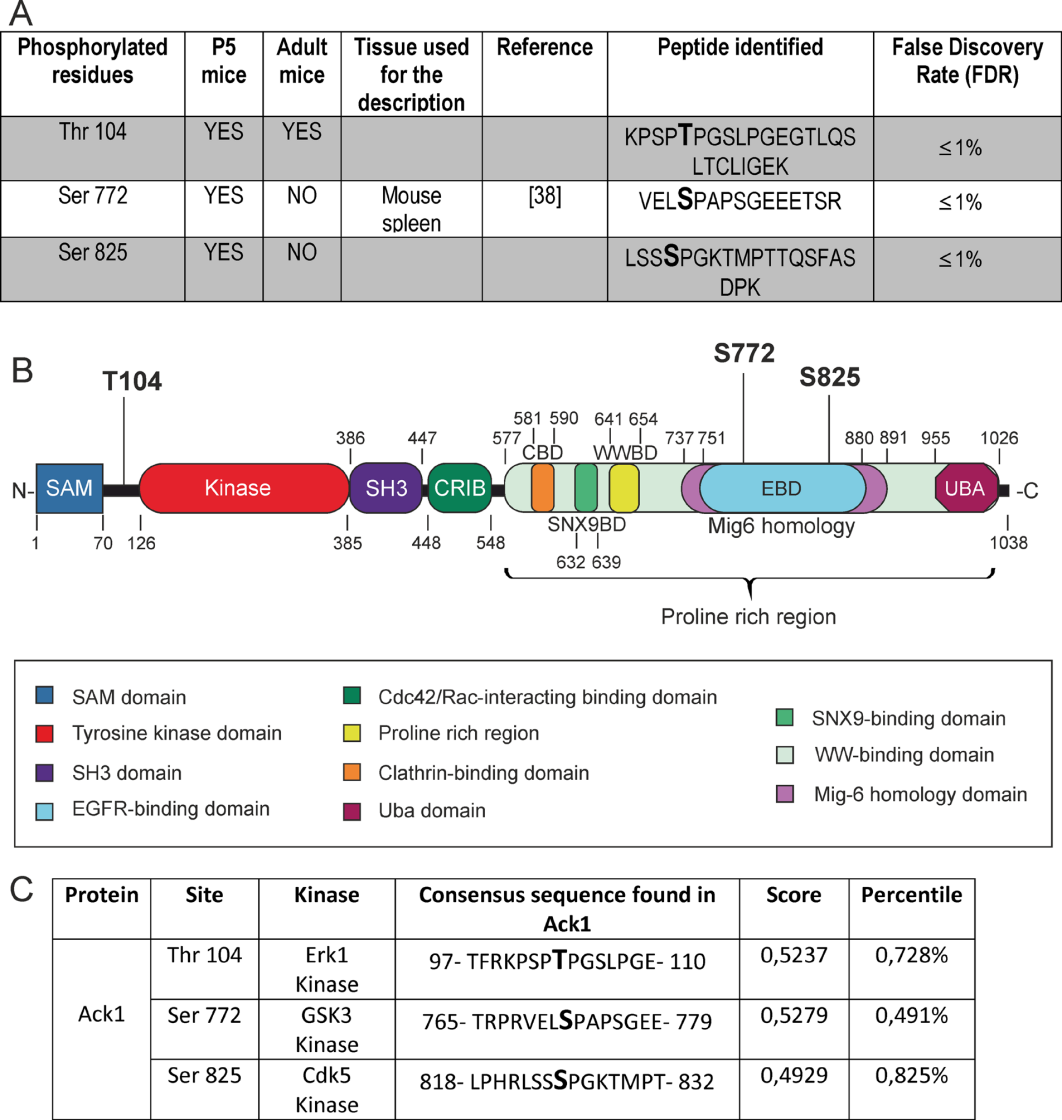
Phosphorylated residues identified in the protein Ack1 (**A**) Summary of the phosphorylated residues found in Ack1. The residues are divided in two groups: enhanced in gray are the phosphorylation residues that have never been previously described *in vivo* by MS, and on the white background those that have. For the latter, the references first describing this phosphorylation are provided. The composition of the peptide isolated in the analysis and the reliability of the assay (shown as a percentile index of false discovery) are also shown. (**B**) Structure of the Ack1 protein. The functional domains are highlighted, as well as the phosphorylated residues identified by LC-MS/MS. (**C**) Putative kinases that could phosphorylate each residue identified, as assigned by an “*in silico*” analysis. The score and percentile indexes of error for the assigned kinases are also specified.

The ionized compounds subjected to LC-MS/MS were resolved in a series of mass/charge ratios, from which the composition of the peptide and the phosphorylated residues were deduced by the software. Two phosphorylated residues in brain tissue, namely Thr 104 and Ser 825, were identified in P5 samples as well as in adult samples. We also identified Ser 772 as a phosphorylated site during brain development (Figure 2A). Supplementary Figure 1 shows the mass/charge ratios found for the peptide in which Thr 104 lies, and the corresponding spectrum.

Both Ser 772 and Ser 825 are located in the Pro-rich region of Ack1, specifically in the EBD comprised in the Mig-6 homology region of the protein. The residue Thr 104 lies between the SAM domain and the catalytic tyrosine kinase domain of Ack1 (Figure 2B).

Furthermore, we performed an “in silico” analysis to evaluate those kinases that potentially phosphorylate these residues. *ScanSite* software revealed particular motifs as strong candidates as substrates of specific protein kinases. The score values and percentiles assigned by the software (see also the corresponding section in Materials and Methods) allow us to conclude that Thr 104 lies in a sequence that matches the consensus sequence of Erk1 substrates; Ser 772 matches the consensus of GSK3 substrates; and Ser 825 matches that of Cdk5 (Figure 2C).

### Co-immunoprecipitation and co-localization of Ack1 with CAMKII-α

**Figure 3:**
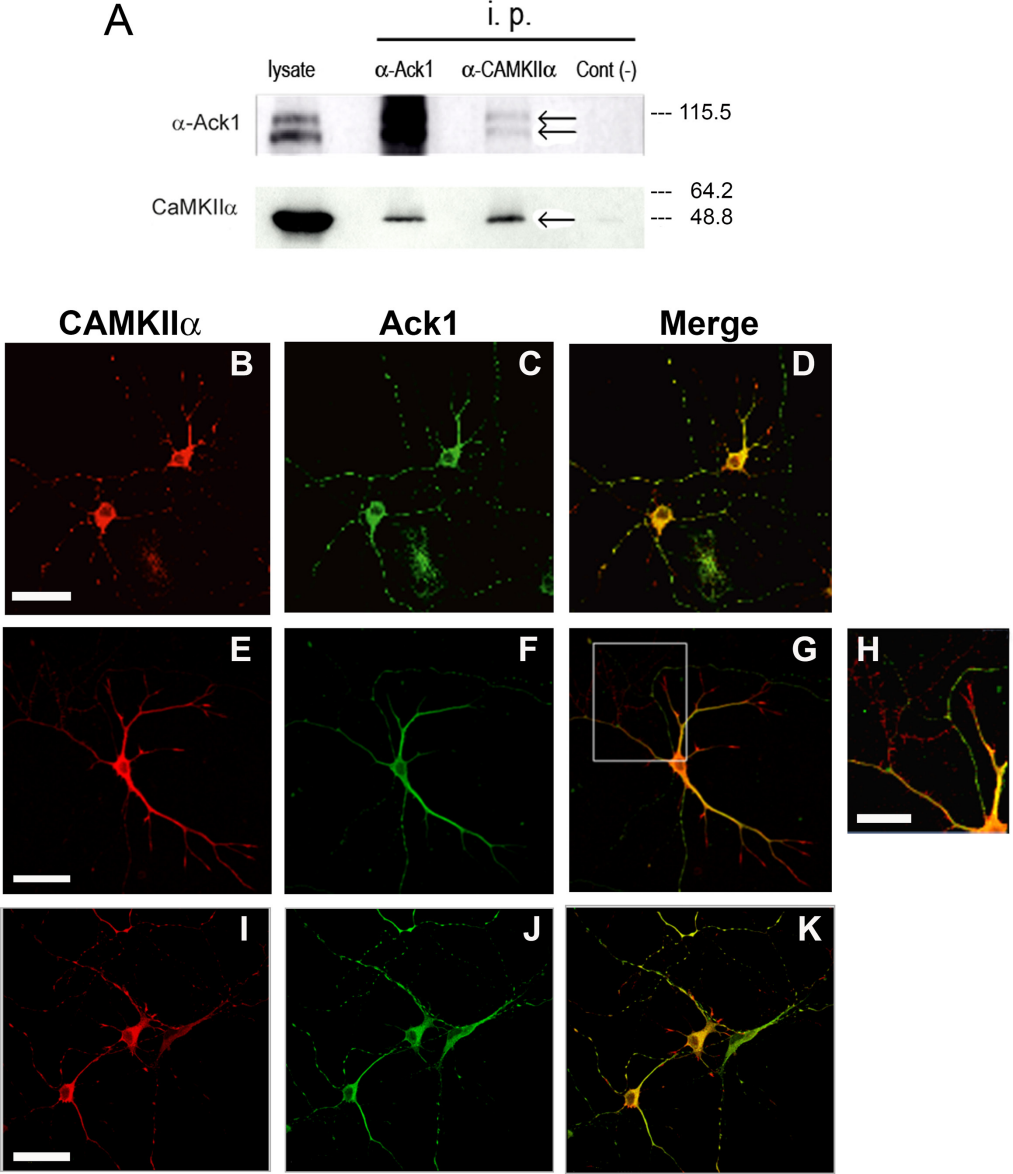
Proteins Ack1 and CAMKII-α coimmunoprecipitate and co-localize (**A**) Homogenized tissue from adult mouse brains was immunoprecipitated with an antibody against Ack1 and an antibody against α-CAMKII, and then analyzed by Western blot with the same antibodies. In the Western blot against α-CAMKII, the True blot secondary antibody was used. Co-immunoprecipitation assays were performed three times with the same results. Right to the blot is shown the molecular weight according to protein ladder. (**B**–**K**) Immunostaining of hippocampal neurons from E16 mouse maintained during 5 days *in vitro*. Ack1 is stained in green and CAMKII-α in red. The images show a high degree of colocalization in B, C, and D, in which two neurons rich in varicosities are shown. Conversely, this colocalization is partial in E, F, and G, as well as in I, J and K. H shows an enlargement of the squared region of G. Scale bar: 40 μm in B–D, 50 μm in E–G and I–K, and 20 μm in H.

CAMKII exerts very relevant functions in synaptic transmission and plasticity [43]. In order to confirm the possible interaction between Ack1 and CAMKII, we performed co-immunoprecipitation assays in adult brain samples followed by Western-blot, which revealed that Ack1 antibodies co-precipitated CAMKII- α; reciprocally, CAMKII-α immunoprecipitates yielded Ack1 in Western blot analyses (Figure 3A). Moreover, co-immunolocalization experiments in hippocampal neuronal cultures showed that CAMKII-α and Ack1 largely colocalized in most neuronal compartments, including soma, dendrites, and axons (Figure 3B–3D). In some cases this co-localization was however partial or incomplete (Figure 3E–3GE, 3I–3KI). In contrast, CAMKII-α was particularly enriched at the tips of developing dendrites and axons (e.g. growth cones), whereas Ack1 was not (Figure 3H). Taken together, these results support the notion that Ack1 and CAMKII-α interact *in vivo* in neurons although not in all compartments.

## DISCUSSION

### Phosphopeptide mapping of Ack1

The catalytic activity of Ack1 increases in parallel with the degree of phosphorylation of the protein [8]. Therefore, description of the phosphorylation residues of this kinase is pivotal for unraveling its functions. However, only the residues Thr 33 [9], Thr113 and Thr 618 [10], Tyr 533 [12], and Ser 622, Ser 789 and Ser 896 [10] have been reported as phosphorylated residues in brain. All of these sites have been identified in adult mouse brain, whereas no residue had ever been described during development, despite the relevance of Ack1 in the developing brain. In this study, we detected the phosphorylation of Ser 772 and Ser 825, both residues located in the EBD, during postnatal brain development. Pro-rich regions are involved in protein-protein interactions, thus possibly modulating various signaling pathways. In particular, the developmental regulation of Ser 772 and Ser 825 phosphorylation in the EBD suggests modulation of Ack1 binding and of signaling to EGFR [14].

The EBD of Ack1 interacts with its own catalytic and SH3 domains, to form an auto-inhibitory conformation of the protein [44]. Supporting this idea, the region spreading from glycine (Gly) residue 786 to Gly 814 of the EBD interacts with the neighboring SH3 domain; and the residues from Gly 814 to Leucine (Leu) 891 of the EBD interact with the catalytic kinase domain of Ack1 [38]. Therefore, Ser 825 is part of the kinase-binding region of the EBD that participates in the auto-inhibitory conformation, suggesting that the phosphorylation of this residue could be concerned with the assembly or disassembly of this structure.

In addition, in P5 and adult brain samples we also detected a phosphorylation site of Ack1 in Thr 104 that lies between the SAM and the catalytic tyrosine kinase domains of the polypeptide. The present study is the first to report the phosphorylation of Thr 104 and Ser 825 of Ack1 in P5 brain. P5 represents a critical development stage of the mouse brain because the first postnatal week is of pivotal importance in dynamic outgrowth, migration, differentiation processes and, especially, synaptogenesis [45, 46]. On the other hand, the phosphorylation of Ser 772 has been previously found in solid tumors of skin melanoma [47] and in spleen [48], in interleukin signaling [49], and in adipocytes [50]. We show for the first time that Ser 722 is phosphorylated during brain development also. Therefore, our data suggest that phosho-Ser 772, phospho-Ser 825, and phospho-Thr 104, are linked to regulatory events in the postnatal and adult brain.

The identification of the residues described here does not rule out further phosphorylated sites because the resolution of the technique we used is constrained by the weight and negative charge of the peptides obtained during trypsinization [25, 51]. Tyr 284 may be such a site, as it is the first described phosphorylation site of the protein [11, 52] but we have not detected it in any sample.

### Putative new partners of Ack1 identified by MS

The description of new partners by LC-MS/MS points to additional functions of Ack1 protein and the pathways in which it can participate. The observation that these proteins were found in at least two independent MS approaches supports the notion that they co-associate with Ack1. This is the case, for instance, of drebrin, CAMKII and α-actinin, among others. Moreover, our immunoprecipitation and colocalization assays further support the interaction between Ack1 and CAMKII. The proposed interacting proteins identified by MS remain to be confirmed as Ack1 partners by additional techniques and procedures.

Most of the putative partners found herein, including drebrin, MAP 6, MAP 4, α-actinin, and the neurofilament medium polypeptide, are involved in cytoskeletal organization (Supplementary Table 1). Ack1 has also been associated with cytoskeletal modulators such as Cdc42 [53] and Dbl [54], suggesting that cytoskeletal regulation in neurons is a key role of this kinase in brain. Both young and adult neurons require an exquisite control of cytoskeletal rearrangement, which is linked to a variety of processes, including migration, axonal growth, synapse formation, and plasticity [55, 56].

Several other Ack1-interacting proteins are directly linked to neurotransmission and physiological plasticity. This is the case, for instance, of the NMDA receptor subunits GRIN1 and GRIN 3, the GTPase activator SynGAP, and the plasticity and physiology regulator CAMKII. CAMKII is well known as a regulator of post-synaptic differentiation and evolution. Our results suggest that these proteins interact both during highly synaptogenic stages such as P5, and in the adulthood, what agrees with a role related to plasticity [43].

As these proteins are known to be tightly regulated by phosphorylation, it is tempting to speculate that the kinase activity of Ack1 targets these major proteins. Our results also suggest that Ack1 protein in neurons is involved in additional cellular processes, including adhesion (because of its association with neurabin-2 or NCAM-I among others) or even vesicle trafficking like SH3-containing grb2-like protein 3-interacting protein 1 [57], these two latter phenomena potentially related to cytoskeletal dynamics. In addition, various metabolism-related mitochondrial proteins (peroxiredoxines 1 and 2, and the precursor of dihydrolipoamide S-acetyltransferase), as well as some regulators of calcium trafficking or RNA processing may also be Ack1 partners. Finally, some of the proteins identified were found only in the P5 brain samples (e.g. NCAM 1 or α-actinin), while others were detected only in the adult brain (e.g. SynGAP, GRIN 3 and CAMKII). It is worth noting that, although the Ack1 phosphopeptides identified in this study do not match with CAMKII consensus, the possibility that Ack1 could phosphorylate CAMKII cannot be ruled out, and it deserves further studies in the near future.

Further analysis of this high variety of putative partners will be performed through Cytoscape network data integration [58], to achieve a better understanding of the biological meaning of this large list of proteins, and to select particular pathways to study. At present, these findings suggest that in neural tissue Ack1 shows developmental stage-specific and –dependent interactions, which could be related to the differential processes that occur in developing and adult neurons.

## MATERIALS AND METHODS

### Animals

Postnatal mice of the OF-1 strain (Charles River, Lyon, France) were used. The day of birth was considered postnatal day 0 (P0). P5 corresponds to animals in their 5 day of postnatal life. Adult animals were 3 months old (P90). All experimental procedures were performed in accordance with the guidelines approved by the Spanish Ministry of Science and Technology, and following the European Community Council Directive 86/609 EEC. In addition, the procedures for animal welfare, anesthesia and sacrificed were approved by the Ethical Committee of Animal Experimentation from University of Barcelona (Numb Ref DAAM 7823).

### Reagents

Polyclonal antibody against Ack1 (384) was generated using the GST fusion proteins corresponding to the Pro-rich region (amino acids 502–1008), as described elsewhere [29]. The monoclonal antibody against Ack1 was generated by Leitat (Barcelona, Spain) and its efficiency has been described previously [18]. As antigen, we used the same GST fusion protein encoding amino acids 502–1008 of Ack1 as for polyclonal antibodies. Mice were injected with the purified protein, and standard procedures were followed to obtain the antibodies [59]. The monoclonal antibody against CAMKII-α was from Affinity Bioreagents (Golden, CO). The goat anti-rabbit-HRP secondary antibody used in the Western Blot analysis was from Sigma (St. Louis, MO), and the antibody against mouse True Blot Ultra WAS from eBioscience (San Diego, CA). Alexa Fluor-488 goat anti-rabbit immunoglobulin G (IgG) (H + L) and Alexa Fluor-546 goat anti-mouse immunoglobulin G (IgG) (H + L) were supplied by Invitrogen (Carlsbad, CA). Protein G-sepharose beads were from Sigma-Aldrich (St. Louis, MO).

### Protein extraction for LC-MS/MS approach

Ten brains were dissected for each P5 assay, and 2 female adult brains (3 months-old) were dissected for each adult assay. The brains were homogenized with a *Polytron* device in a volume of lysis buffer that corresponded to 5 times their weight. Lysis buffer consisted of 50 mM pH 7.5 HEPES (4-(2-hydroxyethyl)-1-piperazineethanesulfonic acid) (Sigma-Aldrich, St. Louis, MO), 150 mM sodium chloride (Panreac, Barcelona, Spain), 1.5 mM magnesium chloride (Sigma-Aldrich, St. Louis, MO), 1 mM EGTA (ethylene glycol tetraacetic acid) (Sigma-Aldrich, St. Louis, MO), 10% glycerol (Millipore, Darmstadt, Germany), 1% Triton X-100 (Panreac, Barcelona, Spain), protease inhibitor cocktail (1×) (Roche, Basel, Switzerland), and the following phosphatase inhibitors: 10 mM tetrasodium pyrophosphate (Sigma-Aldrich, St. Louis, MO); 200 μM sodium orthovanadate (Panreac, Barcelona, Spain); and 10 mM sodium fluoride (Sigma-Aldrich, St. Louis, MO).

The homogenized tissues were left under agitation at 4°C for 20 min. The samples were then centrifuged at 13,000 rpm at 4°C for 20 min, and the supernatants were incubated with a mix of Ack1 monoclonal and polyclonal antibodies at a proportion of 1:1 overnight at 4°C. The next day sepharose beads coupled to protein G (Sigma-Aldrich, St. Louis, MO) were added, and the suspension was incubated for 2 h at 4°C. After this step, the beads were washed 5 times with lysis buffer and resuspended in 1.5 ml of lysis buffer plus 4 volumes of acetone 100% (Panreac, Barcelona, Spain). This resuspension was left overnight at −20°C. The next day, samples were centrifuged at 13,000 rpm at 4°C for 20 min. The pellet was incubated under agitation with 2 volumes of 0.1 M pH 2 glycine (Panreac, Barcelona, Spain) for 5 min at 4°C. The samples were then centrifuged again at 13,000 rpm at 4°C for 5 min, and the supernatants were neutralized with 0.1 volumes of 1 M Tris-HCl pH 8.5 (Panreac, Barcelona, Spain). The samples were then concentrated using 100 KDa Amicon columns (Millipore, Billerica, MA) to achieve a maximum volume of 50 μl.

Finally, the samples were loaded in an 8% polyacrylamide gel (Bio-Rad, Hercules, CA) and subjected to SDS-PAGE electrophoresis. After that, the gel was stained with the fluorescent label *SyproRuby* at 4°C.

### Silver and syproruby staining

Silver staining was performed with Silver Staining kit (Pierce) according to the manufacturer's instructions which consisted in a standard ethanol / acetic acid fixation, ethanol washing, 30’ staining with the reagents supplied, wash in ultrapure water and stop in 5% acetic acid solution.

SyproRuby staining was performed with SYPRO^®^ Ruby protein gel stain kit (Invitrogen, Carlsbad, CA) according to the manufacturer's instructions. Briefly, they consisted in a similar ethanol/acetic acid fixation, SyproRuby staining, and washing. SyproRuby is a fluorescent dye and it was observed on an ultraviolet light transilluminator.

### Mass spectrometry (LC-MS/MS) approach

Bands were cut from the gel and digested with trypsin (Promega (Madison WI)). The result of the digestion was enriched in phosphopeptides by means of a column containing titanium dioxide magnetic beads (GE Healthcare, Barcelona, Spain). The phosphopeptide-enriched fraction was analyzed by LC-MS/MS in a *Nanoacquity* cromatographer (Waters, Cerdanyola del Vallès, Spain) coupled to a LTQ-*OrbitrapVelos* mass spectrometer (Thermo Scientific, Walthman, MA). The spectra obtained from the LC-MS/MS analysis were analyzed using the software *Proteome Discoverer (v1.20)*. Searches were performed using Sequest search engine using *Thermo Proteome Discover* (v.1.3.0.339) against the public Uniprot-SwissProt database. To improve the sensitivity of the database search, Percolator [60] was used to discriminate correct from incorrect peptide spectrum matches. Only peptides reported as high confidence (FDR≤1%) were considered for identification. A score value was assigned to the proteins selected. This value is the result of the sum of the score of all the peptides that match this protein multiplied by the number of peptides found for this protein.

LC-MS/MS was performed six times using adult samples, and two times using P5 samples.

The scores that are shown correspond to the sum of the scores of all the peptides that matched this protein, multiplied by the number of different peptides found for this protein. The value of this initial score is automatically given by the search engine (Sequest in our case) and is subjected to a different algorithm for each protein search. It corresponds to the degree of reliability between the spectrum found and the predicted spectrum for the peptide that best matches the sequence of this protein, with respect to all the trypsin-digested peptides that are found. The higher the score, the more reliable the identification, it means that many peptides that match the protein were found several times

### MALDI-TOF approach

The Matrix-Assisted Laser Desorption/Ionization – Time of Flight (MALDI-TOF) approach was performed by implantation of the peptidic extract in a synapinic acid solid matrix, followed by pulse-irradiation using a nitrogen laser. Score values were also assigned to each peptide found, on the basis of the number of times that a peptide of this protein was found multiplied by the number of peptides found for this protein. The software also assigns a percentile that indicates the percentage of probability that the candidate motif is phosphorylated with respect to all the potential motifs in the protein database. In other words, the database contains thousands of identified substrates and consensus sequences and the greater the agreement with only one particular consensus, the higher the reliability of being phosphorylated by this kinase and not another, and thus the lower the assigned percentile; therefore, a lower percentile indicates major specificity of the candidate motif.

### Analysis “*in silico*” of putative kinases

We used the software *ScanSite* [61] with the parameters of medium stringency. Score values were assigned from the database to the motifs found. Scores closer to 0 correspond to higher similarities with the known consensus for each kinase. A percentile value is also assigned by the software, which indicates the percentile probability for the candidate motif to be phosphorylated by the specific kinase with respect to all potential motifs in the protein database.

### Immunoprecipitation assay and western-blot

A whole brain from an adult mouse was dissected and weighed, and 5 volumes of lysis buffer was added. The lysis buffer used contained 50 mM HEPES (4–2 hydroxyethyl-1-piperazine ethansultanic acid), pH 7.5, 150 mM sodium chloride, 1.5 mM magnesium chloride, 1 mM EGTA (ethylene glycol-bis(2-aminoethylether)-N,N,N′,N′-tetraacetic acid), 10% glycerol, and 1% Triton X-100, containing a protease inhibitor cocktail (1×) (Roche, Basel, CH) and phosphatase inhibitors (10 mM tetra-sodium pyrophosphate, 200 μM sodium orthovanadate, and 10 mM sodium fluoride). The mixture was processed by using a Polytron homogenizer, and insoluble material was removed by centrifugation at 13,000 rpm for 20 min. Next, antibodies were added to 500 μg of total protein from tissue extracts, which were then incubated for 2 h at 4°C. As a control, an anti-GST antibody (Abcam) was used under the same conditions.

Afterwards, sepharose beads coupled to protein G (Sigma-Aldrich, St. Louis, MO) were added, and the suspension was incubated for 1.30 h at 4°C. The beads were theN washed with lysis buffer 5 times and resuspended in 50 μl of lysis buffer and 25 μl of Laemmli loading buffer (25 mM Tris pH 6.5, 0.17 mM β-mercaptoethanol, 2% SDS, 10% glycerol, and 0.0025% bromophenol blue). Next, the samples were analyzed by SDS-PAGE [18], [62].

Samples were run in an 8% polyacrylamide gel at 120 V and transferred to a nitrocellulose membrane in 120 mM glycine, 125 mM Tris, pH 8.5, and 20% methanol. Filters were then saturated in 3% BSA in TBS and incubated with the corresponding antibodies. Protein detection was performed using ECL system (Bio-Rad, Hercules, CA). These co-immunoprecipitation assays were performed three times.

### Primary cultures

Primary cultures of hippocampal neurons were performed as described in [63]. Briefly, the hippocampi of E16 embryos were dissected in PBS-0.6% glucose, trypsinized and plated on poly-D-lysine-coated glass coverslips. The cells were cultured at 37°C in water jacket CO_2_ incubators, during 3–5 days in Neurobasal medium (Thermofisher) devoid of serum and supplemented with B-27 (Thermofisher). These conditions lead to cultures highly enriched in neurons, which contain neurites and varicosities that represent precursors of synaptic contacts.

### Paraformaldehyde fixation and immunofluorescent staining

Cells were fixed with 4% paraformaldehyde in PBS (pH 7.4) for 30 min, permeabilized and blocked with 0.1% Triton X-100 (Sigma-Aldrich, St. Louis, MO) and 10% normal goat serum in PBS for 1 h, washed with PBS, and incubated with primary antibodies (polyclonal Ack1 1:100; monoclonal CAMKII-α 1:100) for 2 h. After being washed with PBS, cells were incubated with both secondary antibodies, namely goat anti-mouse-Alexa Fluor-546 or goat anti-mouse-Alexa Fluor-488 (Invitrogen, Carlsbad, CA), for 1 h. Coverslips were washed with PBS and mounted on glass slides. All procedures were carried out at room temperature [29].

## SUPPLEMENTARY MATERIALS FIGURE AND TABLES









## Abbreviations

Ack1: associated Cdc42 kinase
CAMKII: Calmodulin kinase II
Ser: Serine
Thr: Thronine
Tyr: Tyrosine
