# Supplementary material for: Identification of novel Ack1-interacting proteins and Ack1 phosphorylated sites in mouse brain by mass spectrometry
Source: Oncotarget. 2017 Sep 15;8(60):101146–57. doi: 10.18632/oncotarget.20929 (PMC5731862; doi:10.18632/oncotarget.20929)
Supplement: Supplementary file 1 [file oncotarget-08-101146-s001.pdf]

Identification of novel Ack1-interacting proteins and Ack1 phosphorylated sites in mouse brain by mass spectrometry

SUPPLEMENTARY MATERIALS

A

Sequence: KPSPTPGSLPGEGETLQSLTCLIGEK, T5-Phospho (79.96633 Da), C20-Carbamidomethyl (57.02146 Da)
Charge: +3, Monoisotopic m/z: 883.10675 Da (+0.24 mmu/+0.27 ppm), MH+: 2647.30570 Da, RT: 26.42 min, Identified with: SEQUEST (v1.20); XCorr: 3.52,
Ions matched by search engine: 16/142
Fragment match tolerance used for search: 0.6 Da

Ion Series | Phosphorylation Losses | Neutral Losses | Multiple Neutral Losses | Precursor Ions

#1	b^+	b^{2+}	Seq.	y^+	y^{2+}	#2
1	129.10225	65.05476	K			25
2	226.15502	113.58115	P	2519.21002	1260.10865	24
3	313.18705	157.09716	S	2422.15725	1211.58226	23
4	410.23982	205.62355	P	2335.12522	1168.06625	22
5	591.25383	296.13055	T-Phospho	2238.07245	1119.53986	21
6	688.30660	344.65694	P	2057.05844	1029.03286	20
7	745.32807	373.16767	G	1960.00567	980.50647	19
8	832.36010	416.68369	S	1902.98420	951.99574	18
9	945.44417	473.22572	L	1815.95217	908.47972	17
10	1042.49694	521.75211	P	1702.86810	851.93769	16
11	1099.51841	550.26284	G	1605.81533	803.41130	15
12	1228.56101	614.78414	E	1548.79386	774.90057	14
13	1285.58248	643.29488	G	1419.75126	710.37927	13
14	1386.63016	693.81872	T	1362.72979	681.86853	12
15	1499.71423	750.36075	L	1261.68211	631.34469	11
16	1627.77281	814.39004	Q	1148.59804	574.80266	10
17	1714.80484	857.90606	S	1020.53946	510.77337	9
18	1827.88891	914.44809	L	933.50743	467.25735	8
19	1928.93659	964.97193	T	820.42336	410.71532	7
20	2088.96724	1044.98726	C-Carbami...	719.37568	360.19148	6
21	2202.05131	1101.52929	L	559.34502	280.17615	5
22	2315.13538	1158.07133	I	446.26095	223.63411	4
23	2372.15685	1186.58206	G	333.17688	167.09208	3
24	2501.19945	1251.10336	E	276.15541	138.58134	2

B

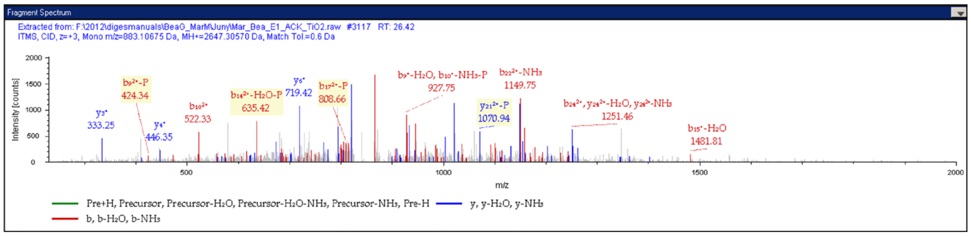

Supplementary Figure 1: (**A**) Table indicating the ratio mass/charge of the ions obtained from the fragmentation of the peptide KPSPTPGSLPGEGETLQSLTCLIGEK, which includes Thr 104 as a phosphorylated residue. (**B**) Spectra obtained from this peptide by LC-MS/MS analysis.

Supplementary Table 1: Proteins identified by LC-MS/MS in the immunoprecipitation products of Ack1 in the different experimental conditions. See Supplementary_Table_1

Supplementary Table 2: Complete list of the proteins identified in an immunoprecipitated sample of P5 mouse brain in one of the LC-MS/MS analyses. See Supplementary_Table_2

Supplementary Table 3: Complete list of the proteins identified in an immunoprecipitated sample of adult mouse brain in one of the LC-MS/MS analyses. See Supplementary_Table_3
