# Supplementary material for: Identification of novel Ack1-interacting proteins and Ack1 phosphorylated sites in mouse brain by mass spectrometry
Source: Oncotarget. 2017 Sep 15;8(60):101146–57. doi: 10.18632/oncotarget.20929 (PMC5731862; doi:10.18632/oncotarget.20929)
Supplement: Supplementary file 2 [file oncotarget-08-101146-s002.docx]

**Supplementary Table 1: Proteins identified by LC-MS/MS in the immunoprecipitation products of Ack1 in the different experimental conditions**

| **PROTEIN** | **NUMBER OF ADULT SAMPLES** | **HIGHEST ADULT SCORE** | **NUMBER OF**  **P5 SAMPLES** | **HIGHEST**  **P5 SCORE** | **FUNCTION** |
| --- | --- | --- | --- | --- | --- |
| Drebrin  AC Q9QXS6 | 6 | 988.50 | 2 | 1278.96 | Actin binding |
| Neurabin-1  AC O35867 | 6 | 36.30 | 2 | 14.75 | Actin binding |
| Neurabin-2  AC Q96SB3 | 6 | 152.54 | 2 | 172.14 | Actin binding |
| Adducin-α  AC Q9QYC0 | 6 | 119.80 | 2 | 53.80 | Actin binding |
| Adducin-β  AC Q9QYB8 | 6 | 75.40 | 2 | 54.27 | Actin binding |
| Spectrin-β  AC Q62261 | 6 | 89.80 | 2 | 32.76 | Actin binding |
| Spectrin-α  AC P02549 | 5 | 160.91 | 2 | 135.40 | Actin binding |
| Ankyrin-2  AC Q8C8R3 | 5 | 22.03 | 2 | 83.71 | Actin binding |
| Myosin-10  AC P35580 | 5 | 546.77 | 2 | 361.90 | Actin motor |
| Actinin-α  AC P12814 | 5 | 656.12 | --- | --- | Actin binding |
| Synaptopodin  AC Q8CC35 | 5 | 107.34 | --- | --- | Actin binding & PSD |
| Cadherin-2  (N-Cadherin)  AC P15116 | 5 | 59.08 | --- | --- | Adhesion  & actin binding |
| Catenin-α2  AC Q61301 | 6 | 265.11 | 2 | 79.88 | Actin binding |
| Catenin-β  AC P35222 | 6 | 135.05 | 2 | 35.56 | Actin binding  & signal transduction |
| Catenin-δ2  AC O35927 | 6 | 43.29 | 2 | 23.19 | Actin binding  & signal transduction |
| MAP-6  AC Q7TSJ2 | 6 | 320.88 | 2 | 14.23 | MT binding |
| MAP-4  AC P27546 | 5 | 31.29 | 2 | 30.72 | MT binding |
| MAP-7  (Enscosin)  AC Q3TRR0 | 4 | 8.27 | --- | --- | MT binding |
| Kinesin heavy ch. 5C  AC P28738 | 6 | 313.83 | 1 | 13.82 | MT motor |
| Neurofilament medium polyp  AC P08553 | 6 | 195.55 | 2 | 60.82 | IF |
| Neurofilament light polyp.  AC P08551 | 6 | 492.33 | 1 | 166.68 | IF |
| Internexin-α  AC P46660 | 5 | 183.91 | 1 | 244.98 | IF |
| Plakophilin-4  AC Q68FH0 | 5 | 75.48 | 1 | 19.07 | IF binding |
| NCAM-1  AC P13596 | 4 | 49.82 | 1 | 2.90 | Adhesion |
| AP2 complex,  Α1 subunit  AC P17427 | 4 | 57.82 | --- | --- | Clathrin-mediated endocytosis |
| Contactin  AC P22063 | 4 | 178.58 | --- | --- | Adhesion |
| ERC protein 2  AC O15083 | 5 | 86.27 | --- | --- | Nerve term cytomatrix |
| Matrin-3  AC Q8K310 | 6 | 10.21 | 2 | 48.64 | Nuclear matrix |
| Ack1  AC O54967 | 4 | 35.39 | 1 | 11.35 | Signaling |
| Rho GEF 2  AC Q60875 | 5 | 15.09 | --- | --- | Signaling |
| CAM K II α  AC P11798 | 6 | 376.25 | 1 | 32.29 | Signaling |
| CAM K II β  AC P08413 | 6 | 322.32 | 1 | 74.42 | Signaling |
| SH3-cont Grb2-like interact prot 1  AC Q8VD37 | 5 | 95.25 | 1 | 7.42 | Signaling |
| SynGAP (Ras GAP)  AC F6SEU4 | 5 | 253.51 | --- | --- | Signaling at the synapse |
| SLIT/ROBO  Rho GAP2  AC O43295 | --- | --- | 2 | 39.27 | Signaling in migration |
| Src kinase signal inhibitor 1  AC Q9QWI6 | 4 | 84.18 | 1 | 8.11 | Signaling |
| Connector enhancer of kinase sup Ras 2  AC Q80YA9 | 5 | 57.62 | --- | --- | Signaling |
| Discs large homol prot 1  AC Q811D0 | 5 | 52.70 | --- | --- | Signaling  (Synapse scaffolding) |
| Discs large homol prot 2  AC Q63622 | 5 | 132.94 | 1 | 29.71 | Signaling (guanylyl cyclase & scaffolding) |
| Discs large homol prot 3  AC Q92796 | 4 | 53.61 | 1 | 8.00 | Signaling (guanylyl cyclase & scaffolding) |
| Discs large homol prot 4  AC P31016 | 6 | 621.59 | 1 | 9.33 | Signaling  (NMDA scaffolding) |
| Prot phosphatase 1 reg sub 12A  AC Q9DBR7 | 4 | 39.04 | 1 | 34.06 | Signaling  (inhibitor of PP1) |
| Sorbin and SH3 domain-cont prot 1  AC Q62417 | 6 | 37.19 | --- | --- | Signaling (Actin dynamics regulation) |
| UDP-N-Acetyl-glucosamine transfer  AC A9IXV7 | 4 | 23.54 | --- | --- | glycosylation |

The proteins identified here have been assigned a reliability score (see the corresponding section in Material and Methods). “AC” in the first column correspond to the Accession Code for the proteins found.

This table shows the proteins that were identified in at least four independent experiments out of the six adult assays, and / or at least one of the P5 assays. Thus, the “Number of samples” correspond to distinct assays performed in samples from distinct animals. The corresponding columns report the number of times that a particular protein was identified, as well as the highest score obtained. A brief comment on the main cellular significance of every protein found is also included.

A score value was assigned to the proteins selected. This value is the result of the sum of the score of all the peptides that match this protein multiplied by the number of peptides found for this protein. Because the proteins here shown were detected in many assays, only the highest score found is reported.
